# Obituary

**Published:** 1851-11

**Authors:** 


### OBITUARY.

Died, on the 16th of Oct., Nichols Hard, M. D., Prof. of Anatomy in the University of Iowa. Prof. Hard maintained a good character as a pleasing and instructive lecturer during his connection with the Medical schools at Laporte, Ind., and Keokuk, Iowa, and enjoyed a high reputation as a practitioner in Aurora, Ills., the place of his residence. He has been cut down in the prime of life and in the midst of his usefulness.

—On the 30th of Oct., at Oswego, Ills., of Typhoid Fever, Dr. Isaac Ives.

—At Carlinville, Ills., on the 16th of Sept., of Cholera, Dr. Edward Wright.

—Dr. Wright graduated at the last commencement of Rush Medical College. He was a young man of excellent character and gave unusual promise of attaining to eminence in his profession.

